# Structural insights into multitargeting *Mycobacterium tuberculosis* Pkn kinases

**DOI:** 10.1128/spectrum.00049-26

**Published:** 2026-07-14

**Authors:** Angelika Pölläniemi, Ya Miao, Lotta Laitila, Hannaleena Piippo, Milka Hammarén, Mataleena Parikka, Teemu Haikarainen

**Affiliations:** 1 Faculty of Medicine and Health Technology, Tampere University 101287, Tampere, Finland; 2 European Molecular Biology Laboratory 9471, Heidelberg, Germany; 3 Fimlab Laboratories, Tampere, Finland; Griffith University - Gold Coast Campus, Queensland, Australia

**Keywords:** antimycobacterial, drug discovery, X-ray crystallography, Pkn kinases

## Abstract

Tuberculosis remains one of the leading major global health challenges, driven by the emergence of multidrug-resistant and extensively drug-resistant bacterial strains. Resistant strains complicate treatment, which often requires prolonged use of toxic second- and third-line drugs. Protein phosphorylation plays critical roles in *Mycobacterium tuberculosis*, with serine/threonine kinases PknA, PknB, and PknG being essential for survival, virulence, and persistence. In this study, we screened an in-house kinase inhibitor library to identify compounds targeting these kinases. Four structurally diverse hits from the screening inhibiting all three kinases *in vitro* were selected for further analysis. Hits were evaluated for their ability to inhibit *M. tuberculosis* growth and characterized structurally using X-ray crystallography, molecular docking, and isothermal titration calorimetry. Our findings provide a structural framework for the development of multitargeting kinase inhibitors, offering a potential strategy to combat drug-resistant *M. tuberculosis*.

**IMPORTANCE:**

Drug-resistant tuberculosis is a growing global health threat that is increasingly difficult to treat with existing antibiotics, necessitating the discovery of new therapeutic strategies. This study focuses on protein kinases, key regulatory enzymes that help *Mycobacterium tuberculosis* survive, cause disease, and persist in the host. By identifying small molecules that can simultaneously block multiple essential kinases, this work introduces a promising multitarget approach to combat tuberculosis. Using structural and biophysical methods, we reveal how these compounds interact with their targets, providing a clear blueprint for improving their effectiveness. These insights advance the rational design of next-generation antitubercular drugs and open new avenues for tackling multidrug- and extensively drug-resistant tuberculosis.

## INTRODUCTION

Tuberculosis (TB) is the leading cause of death from a single infectious agent worldwide, surpassing HIV and COVID-19 in recent years. In 2024, TB caused approximately 1.23 million deaths and an estimated 10.7 million new cases, underscoring its persistent global health burden despite decades of control efforts (1–4). Currently, it is predicted that around one-third of the world’s population is latently infected with the pathogen *Mycobacterium tuberculosis*, and 5–10% of these individuals will go on to develop active TB (5). The spread of multidrug-resistant and extensively drug-resistant *M. tuberculosis* strains is particularly concerning (6), with nearly 390,000 multidrug-resistant tuberculosis (MDR-TB) cases reported in 2024 (4). Treating resistant disease forms requires the combination of second- and third-line drugs that are more toxic, less effective, and have a long duration of treatment (up to 18 months or longer) (7). In cases where the disease progresses into totally drug-resistant TB, it becomes essentially untreatable.

Protein phosphorylation is a fundamental signaling mechanism that regulates cellular functions in response to external signals. In many well-researched bacteria, two-component systems are the main form of phosphorylation-based signal transduction (8). However, in other bacteria, such as *M. tuberculosis* and other Actinobacteria, Ser/Thr and Tyr protein kinases also contribute to transmembrane signal transduction (9, 10). Pkn kinases are a family of 11 serine/threonine protein kinases in *M. tuberculosis*. Two Pkn kinases, protein kinase A (PknA) and protein kinase B (PknB), are essential for *M. tuberculosis* (11, 12)*,* while protein kinase G (PknG) is critical for virulence and plays a key role in bacterial persistence within the host (13–15). PknA is important for mycobacterial survival in different growth conditions. The essentiality of PknA arises from its participation in the regulation of several critical cellular processes, such as cell division, growth, and morphology (11, 12). PknB is considered a highly prospective target for the therapeutic intervention of TB due to its role in cell division, biofilm formation, cellular metabolism, and resuscitation processes (16). PknG, the only cytosolic kinase secreted into host macrophages, functions as a virulence factor by inhibiting phagosome–lysosome fusion, thereby enhancing intracellular survival (17). Additionally, it supports metabolic adaptation and promotes the persistence of non-replicating mycobacteria (18).

PknB has received attention as a drug target, which has led to the discovery of several PknB inhibitors. However, a poor correlation between PknB IC_50_ and anti-*M*. *tuberculosis* MIC has been previously observed (19–21). In certain cases, reducing compound lipophilicity has significantly improved the antibacterial activity, potentially by increasing membrane permeability of the compounds (22). Interestingly, a selective PknG inhibitor, although not affecting bacterial growth *in vitro*, led to phagosome–lysosome fusion in infected macrophages and in the death of internalized mycobacteria (14, 23). The effect of PknG inhibition in the intracellular survival of *M. tuberculosis* has also been observed with other inhibitor scaffolds, leading to decreased intracellular survival of *M. tuberculosis* in infected macrophages (24).

Simultaneous inhibition of the three Pkn kinases could lead to effective inhibition of mycobacterial growth with a low propensity for resistance development (25). Multitargeting reduces the likelihood that a point mutation in one gene will confer clinically meaningful resistance because effective escape would require coordinated mutations across three distinct kinase genes. Targeting multiple essential kinases at once therefore increases the genetic barrier to resistance and can produce synergistic bactericidal effects that are harder for the pathogen to overcome.

**Fig 1:**
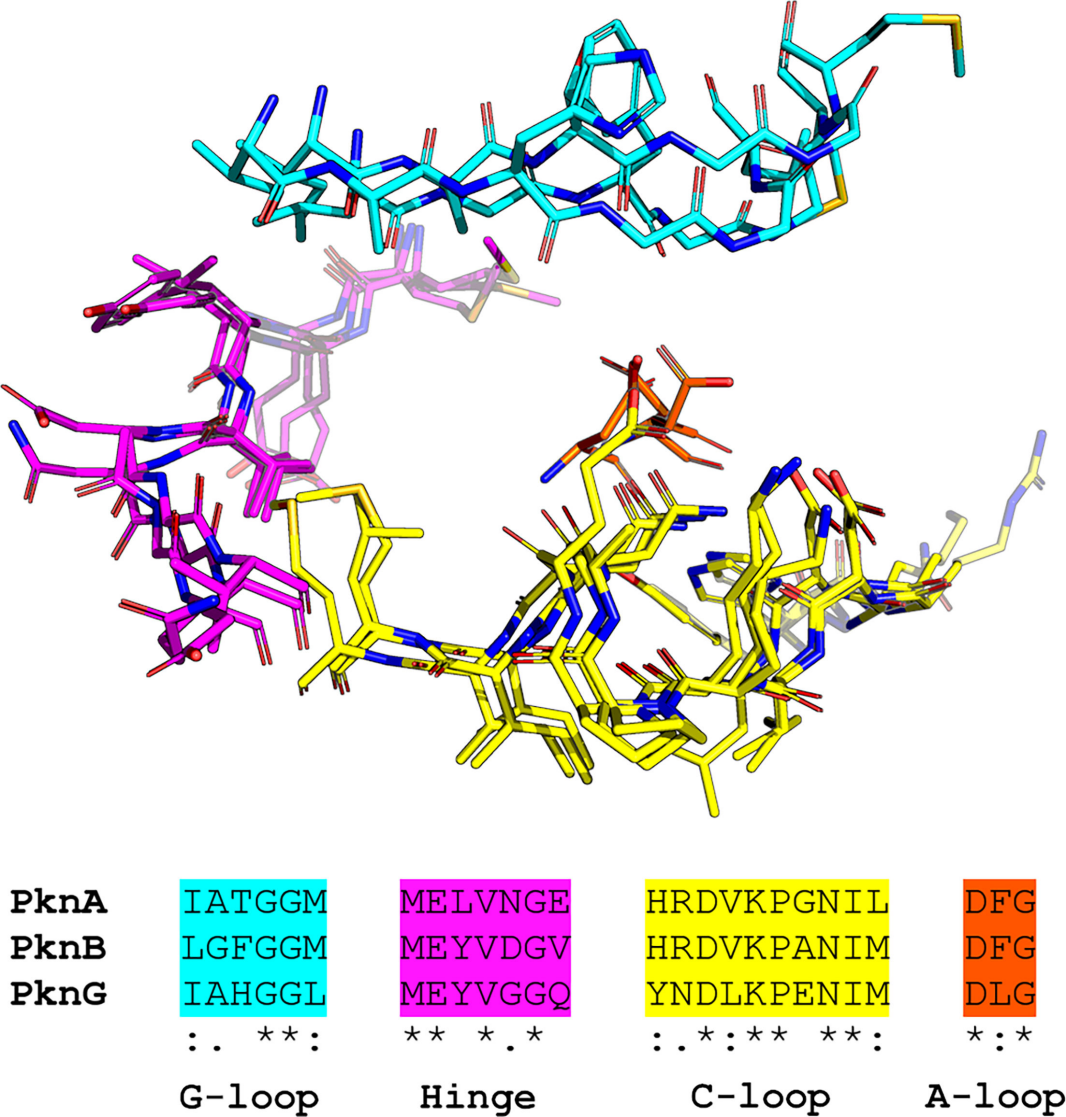
Comparison of ATP-binding pockets of PknA, PknB, and PknG. The figure shows the regions around ATP-binding pocket critical for inhibitor binding. Fully conserved residues are indicated by an asterisk (*), highly similar residues with a colon (:), and weakly similar residues with a period (.). G-loop: glycine-rich loop; C-loop: catalytic loop; A-loop: activation loop.

The only conserved domain across the essential Pkn kinases is the kinase domain, although the kinase domains of PknA, B, and G do not share a very high conservation as their sequence similarities range from 21 to 39%. Thus, designing inhibitors with broader specificity may increase the off-targeting risk with host kinases. Still, the critical residues for inhibitor binding around the ATP-binding pocket show higher conservation (Fig. 1). To identify potential inhibitor scaffolds for the development of multitargeting Pkn inhibitors, we screened an in-house kinase inhibitor library for potential Pkn inhibitors. The obtained hits were evaluated for their ability to inhibit *M. tuberculosis* growth *in vitro*. The binding modes of the hit compounds to Pkn kinases were characterized by X-ray crystallography and molecular docking, while interaction mechanisms were characterized by isothermal titration calorimetry (ITC). The results provide a structural framework that supports the development of new antimycobacterial compounds.

## RESULTS

### Inhibitor screening and effect on bacterial growth

**Fig 2:**
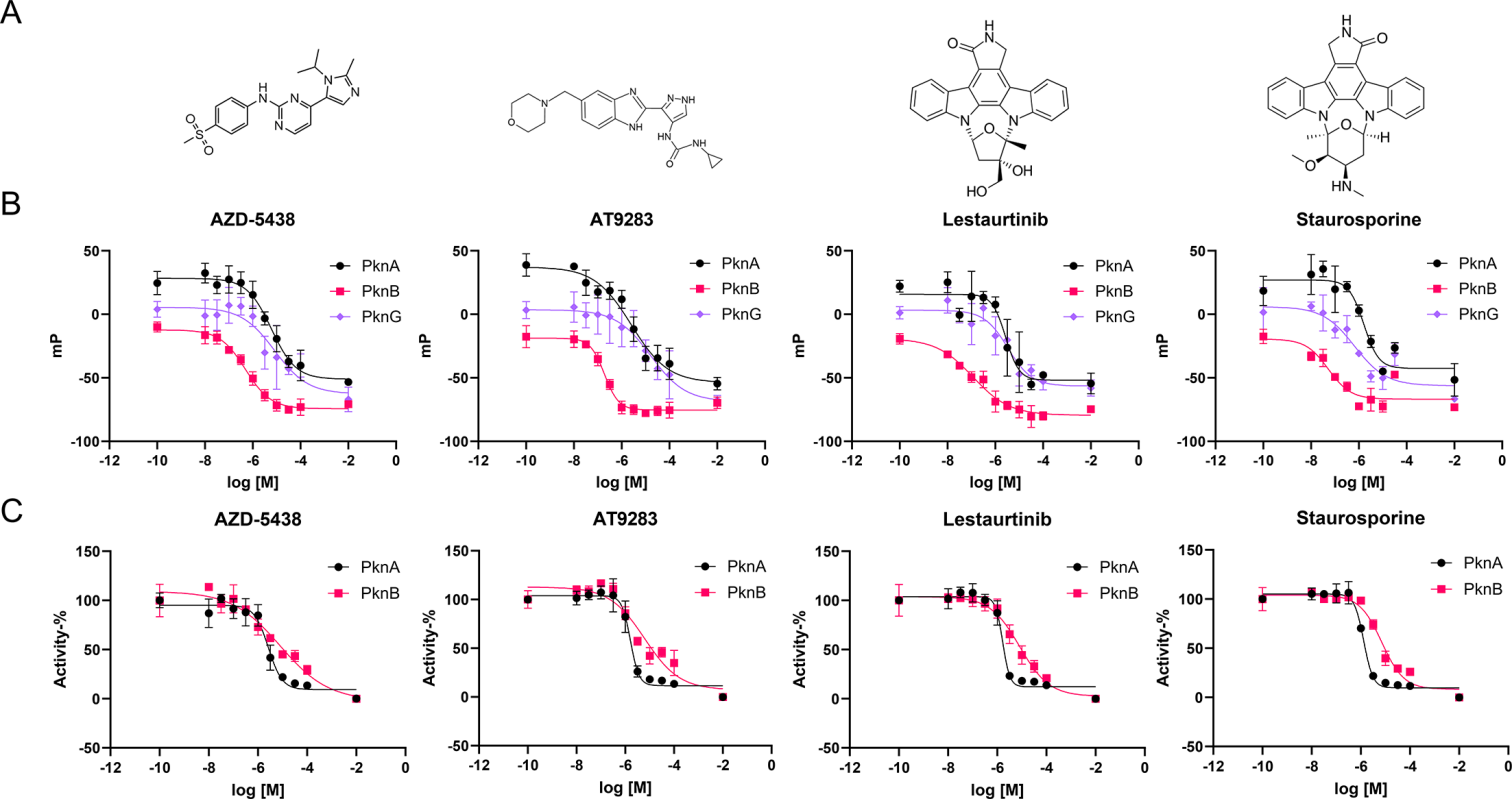
*In vitro* assay results. (**A**) Structures of the compounds. (**B**) Dose-response (IC_50_) curves of the tested compounds against individual kinases, measured with fluorescence polarization (*n* = 1); bars represent standard deviation of a triplicate measurement. mP, millipolarization. (**C**) IC_50_ curves of the compounds with PknA and PknB, measured with the kinase activity assay (*n* = 1); bars represent standard deviation of triplicate measurements.

To facilitate the identification of multitargeting Pkn kinase inhibitors, we optimized an *in vitro* activity-based kinase screening assay for recombinant PknA protein. The assay utilizes a fluorescence-based readout, which correlates with the ATP consumption in the kinase reaction. We employed an in-house kinase-targeted small-molecule library to screen for PknA inhibitors. The screening was carried out as a single-point measurement at 10 µM compound concentration, yielding 23 hits (Fig. S1) that inhibited JAK2 activity by at least 50%. To account for potential false positives arising from inhibition of the assay components, the hits were re-tested with the activity assay without the kinase (data not shown). None of the hits were identified as false positives, and four most potent compounds were chosen for further analysis. These compounds are AZD-5438, AT9283, lestaurtinib, and staurosporine (Fig. 2A). The compounds or their analogs are known clinical-stage kinase inhibitors primarily targeting Janus kinase 2 (JAK2) and/or cyclin-dependent kinase 2 (CDK2). Specifically, AZD-5438 inhibits CDK1, CDK2, and CDK9 (26) with nanomolar potencies. AT9283 is a potent inhibitor of Aurora kinase A and B, JAK2, and Abl (T315I), also at nanomolar concentrations (27). Lestaurtinib is a nanomolar-range receptor protein tyrosine kinase (RPTK) inhibitor targeting JAK2, FLT3, and TrkA (28–30). Staurosporine, a well-known pan-kinase inhibitor, broadly inhibits kinases including PKA, PKC, c-Fgr, v-SRC, and ERK1 (31). The IC_50_ values of the main human targets are listed in Table S1.

Next, we measured the IC_50_ values of the hits. The IC_50_ value is the concentration of a compound required to reduce target activity by 50%, and it is measured to quantify the potency of an inhibitor and enable comparison between compounds. The IC_50_ values were determined for all three Pkn kinases (PknA, PknB, and PknG) with all compounds, utilizing the activity assay. PknG did not exhibit sufficiently high activity levels in the assay. Therefore, we utilized a competition fluorescence polarization (FP) assay to measure protein-ligand binding interactions for PknG potency measurements. FP utilizes a fluorescently labeled ATP-competitive kinase inhibitor as a tracer and was also used as an orthogonal method when evaluating PknA and PknB inhibition. The IC_50_ results for all kinases are shown in Fig. 2B and C and in Table 1. The hits showed consistent kinase inhibition against all three targets, with IC_50_ values mostly in the lower micromolar range with both methods. PknB was the most potently inhibited kinase with submicromolar IC_50_ values. The protein concentration used for IC_50_ determination likely limited the measurable range of the assay. IC_50_ values below half of the protein concentration cannot be reliably estimated, which prevented accurate determination of the true IC_50_ in most cases, particularly in the activity assay.

**TABLE 1:** IC_50_ values, MICs, and molecular properties of the hits

	AZD-5438		AT9283		Lestaurtinib		Staurosporine	
	IC_50_ (µM)	pIC_50_ ± SEM	IC_50_ (µM)	pIC_50_ ± SEM	IC_50_ (µM)	pIC_50_ ± SEM	IC_50_ (µM)	pIC_50_ ± SEM
Activity assay								
PknA	2.6	5.6 ± 0.07	1.6	5.8 ± 0.05	1.7	5.8 ± 0.04	1.3	5.9 ± 0.03
PknB	8.2	5.1 ± 0.19	6.2	5.2 ± 0.17	8.2	5.1 ± 0.10	7.1	5.2 ± 0.08
PknG	ND^*a*^	ND	ND	ND	ND	ND	ND	ND
Fluorescence polarization								
PknA	6.0	5.2 ± 0.11	2.5	5.6 ± 0.16	2.7	5.6 ± 0.12	1.6	5.8 ± 0.12
PknB	0.49	6.3 ± 0.08	0.19	6.7 ± 0.06	0.12	6.9 ± 0.15	0.05	7.3 ± 0.20
PknG	8.3	5.1 ± 0.27	16.4	4.8 ± 0.25	3.4	5.5 ± 0.18	0.66	6.2 ± 0.28
MICs (µM)								
After 7 days	100		100		100		>100	
After 14 days	100		100		50		>100	
Molecular properties (rule of five)								
XLogP3	2.2		0.5		2.2		3.2	
H-bond donors	1		4		3		2	
H-bond acceptors	6		5		4		4	
Molecular weight	371.5		381.4		439.5		466.5	

^*a*^ ND, not determined.

Due to promising *in vitro* activity, we assayed the hit compounds in mycobacterial cells. We utilized the avirulent *M. tuberculosis* strain H37Ra in a broth dilution assay using two known anti-tuberculosis drugs, isoniazid and rifampicin, as positive controls. The minimal inhibitory concentrations of the hits were analyzed after 7 and 14 days (Table 1 and Fig. S2). AZD-5438, AT9283, and lestaurtinib prevented bacterial growth at 100 µM concentration, whereas staurosporine did not fully inhibit growth at 100 µM. At the 14-day time point, lestaurtinib at concentrations of 50 µM and above prevented bacterial growth. The other compounds did not show a difference in bacterial growth compared to the time point on day 7.

### Inhibitor binding thermodynamics

Due to limitations of the screening assay in potency determination and to gain mechanistic insights into inhibitor binding, we used ITC to measure the binding of the compounds to the three Pkn kinases. ITC measures the change in heat upon molecular binding events, which can be used to determine thermodynamic parameters and binding affinity. As expected, several compounds displayed higher binding affinities compared with the activity and FP assays (Table 2 and Fig. S3). In general, PknB was the most potently inhibited kinase by the compounds, and all four hits displayed submicromolar affinities toward PknB. The planar and rigid staurosporine and its analog lestaurtinib displayed the lowest overall affinity toward the kinases. Notably, the binding of these compounds was mainly driven by entropy, and the minimal enthalpic contribution prevented their analysis with PknA (both staurosporine and lestaurtinib) and PknG (staurosporine). In contrast, the binding of AZD-5438 and AT9283 was driven by enthalpy with a strong opposing entropy for PknA and PknB. Interestingly, PknG displayed a more balanced thermodynamic signature for these compounds with a positive entropy contribution. AT9283 was the most potent inhibitor displaying submicromolar binding affinities toward all three kinases. In summary, the thermodynamic signature of inhibitor binding was similar between PknA and PknB, both displaying enthalpy-driven mechanisms for the most potent compounds (AZD-5438 and AT9283). PknG, in contrast, exhibited positive entropy contributions for the compounds.

**TABLE 2:** Analysis of inhibitor binding thermodynamics^*a*^

	ΔG (kcal/mol)	ΔH (kcal/mol)	−TΔS (kcal/mol)	Kd (nM)	*n*
PknA					
AZD-5438	−8.2	−13.4 ± 0.5	5.2	940	0.5
AT9283	−9.3	−15.7 ± 0.1	6.4	160	0.9
Lestaurtinib	ND^*b*^	ND	ND	ND	ND
Staurosporine	ND	ND	ND	ND	ND
PknB					
AZD-5438	−8.7	−10.7 ± 0.1	2.0	390	0.5
AT9283	−10.3	−21.7 ± 0.2	11.4	30	0.7
Lestaurtinib	−9.3	−4.7 ± 0.1	−4.6	150	1.3
Staurosporine	−9.0	−3.0 ± 0.1	−6.0	250	0.6
PknG					
AZD-5438	−7.7	−4.3 ± 0.3	−2.8	2,000	1.1
AT9283	−8.8	−6.1 ± 0.2	−2.8	340	1.0
Lestaurtinib	−7.5	−1.0 ± 0.1	−6.5	3,000	2.7
Staurosporine	ND	ND	ND	ND	ND

^*a*^ ITC measurements were performed at 25°C with protein concentrations between 5 µM and 20 μM and inhibitor concentrations between 10 µM and 200 μM.

^*b*^ ND, not determined.

### Structural basis for inhibitor binding

**Fig 3:**
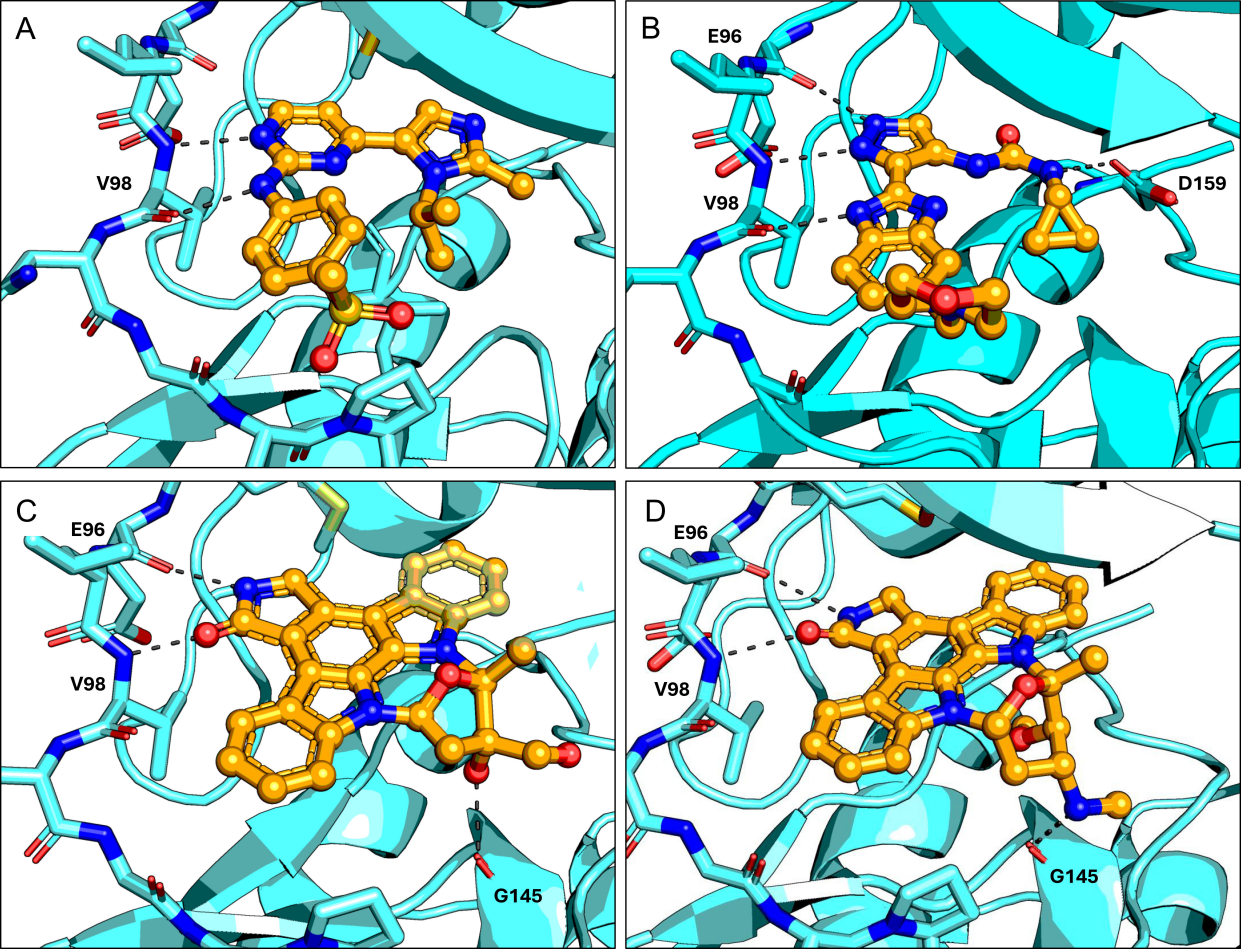
Experimental crystal structures of PknA in complex with the hit compounds. The residues that form bonds have been labeled; black dashed lines indicate hydrogen bonds. (**A**) AZD-5438 (1.70 Å, PDB ID: 9TMX). (**B**) AT9283 (1.45 Å, PDB ID: 9TN5). (**C**) Lestaurtinib (2.13 Å, PDB ID: 9TMQ). (**D**) Staurosporine (1.90 Å, PDB ID: 9TMR).

To analyze the kinase-inhibitor interactions in detail, we crystallized the four inhibitors with PknA. As expected from the FP-based ATP-displacement assay, all compounds bind to the ATP-binding site of the kinase. The compounds anchor to the hinge region of the pocket, forming two or three hydrogen bonds with the hinge (Fig. 3 and Fig. S4). AT9283 is the only compound forming three hydrogen bonds to the hinge, which likely explains its high enthalpy gain upon binding (Fig. 3A). The compound interacts with the backbone carbonyl of Glu96 and makes two hydrogen bonds with the backbone amide nitrogen and carbonyl oxygen of Val98. Additionally, AT9283 forms an additional hydrogen bond with Asp159 from the DFG motif in the catalytic loop. AZD-5438 is stabilized by two hydrogen bonds to the backbone amide nitrogen and carbonyl oxygen of Val98 (Fig. 3B). Staurosporine and lestaurtinib exhibit similar coordination, anchoring to the hinge via hydrogen bonds with the backbone carbonyl of Glu96 and the backbone amide nitrogen of Val98 (Fig. 3C and D). Both compounds also form an additional hydrogen bond with Gly145 in the catalytic loop.

**Fig 4:**
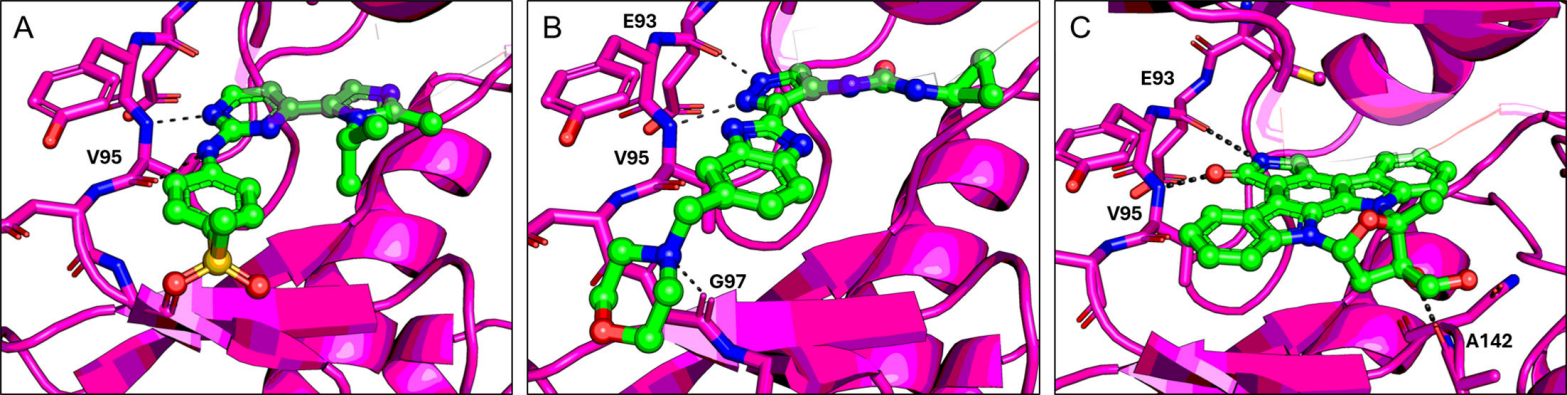
Docking of the hit compounds to PknB. Residues involved in bonding are labeled, and hydrogen bonds are shown as black dashed lines. (**A**) AZD-5438. (**B**) AT9283. (**C**) Lestaurtinib.

**Fig 5:**
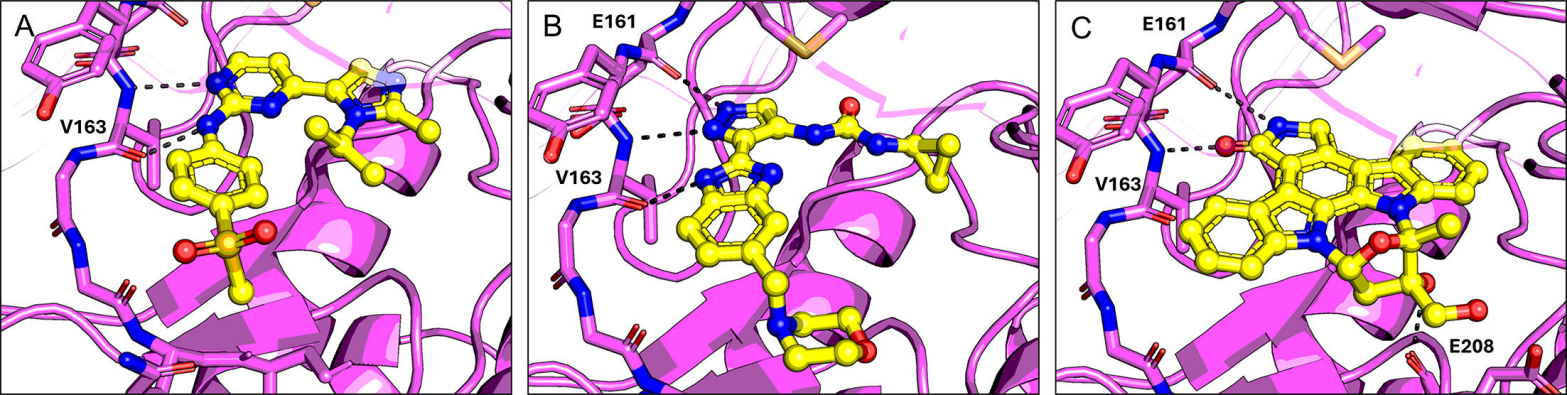
***.*** Docking of the hit compounds to PknG. Residues involved in bonding are labeled, and hydrogen bonds are shown as black dashed lines. (**A**) AZD-5438. (**B**) AT9283. (**C**) Lestaurtinib.

To gain structural insights into inhibitor binding to PknB and PknG, we docked the compounds to their ATP-binding pockets ( Fig. 4 and 5). Importantly, the docking poses of the compounds were highly similar to PknA crystal structures, indicating similar binding modes. For AZD-5438, the docking poses in PknB (Fig. 4A) and PknG (Fig. 5A) reproduced the same hinge-region hydrogen-bonding interactions observed in the PknA crystal structure. In contrast, AT9283 exhibited subtle but notable differences between its docked and experimental conformations. When docked to PknB (Fig. 4B), the benzimidazole moiety was tilted upward, and the pyrazole ring maintained two hinge hydrogen bonds, while the benzimidazole lacked direct interactions. Instead, the morpholino nitrogen formed a hydrogen bond with a hinge glycine, an interaction absent in the PknA structure, although this pose is likely unstable due to the flexible nature of the linked morpholino group. For PknG (Fig. 5B), AT9283 preserved the hinge interactions seen in PknA, but the hydrogen bond to Asp in the DFG motif was lost, likely due to the DLG variant in PknG altering the motif’s backbone conformation. Docking of lestaurtinib revealed similar binding modes for PknB (Fig. 4C) and PknG (Fig. 5C), although in PknA the compound was positioned closer to the methionine gatekeeper, possibly reflecting gatekeeper conformational differences. Finally, the docking of staurosporine to PknB and PknG was unsuccessful, potentially reflecting the weak enthalpic contribution to binding, as direct electrostatic interactions could not be established during docking.

### Comparison of inhibitor binding modes to human kinases

All hit molecules are known human kinase inhibitors, and we sought to compare the binding modes between mycobacterial and human kinases to identify regions for improving compound selectivity. We superposed the PknA structures with crystal structures of the hits in complex with human kinases such as Janus kinase 2 (JAK2) and cyclin-dependent kinase 2 (CDK2). JAK2 is a non-receptor tyrosine kinase and regulates pathways related to hematopoiesis, immune responses, and cell growth (32). CDK2 is a serine/threonine kinase and a key regulator of the G1/S transition, S-phase, and G2/M transition of the cell cycle (33).

The binding of lestaurtinib to PknA, a clinical JAK2 inhibitor, was compared to the JAK2-lestaurtinib complex (PDB ID: 8BPW) (34). AT9283 has also been identified as a JAK2 inhibitor, and to analyze the binding modes between PknA and JAK2, we determined the crystal structure of the JAK2–AT9283 complex. The binding modes of the CDK2 inhibitor AZD-5438 and pan-kinase inhibitor staurosporine were analyzed between PknA and CDK2 (PDB ID: 6GUH [35] and 4ERW [36], respectively).

**Fig 6:**
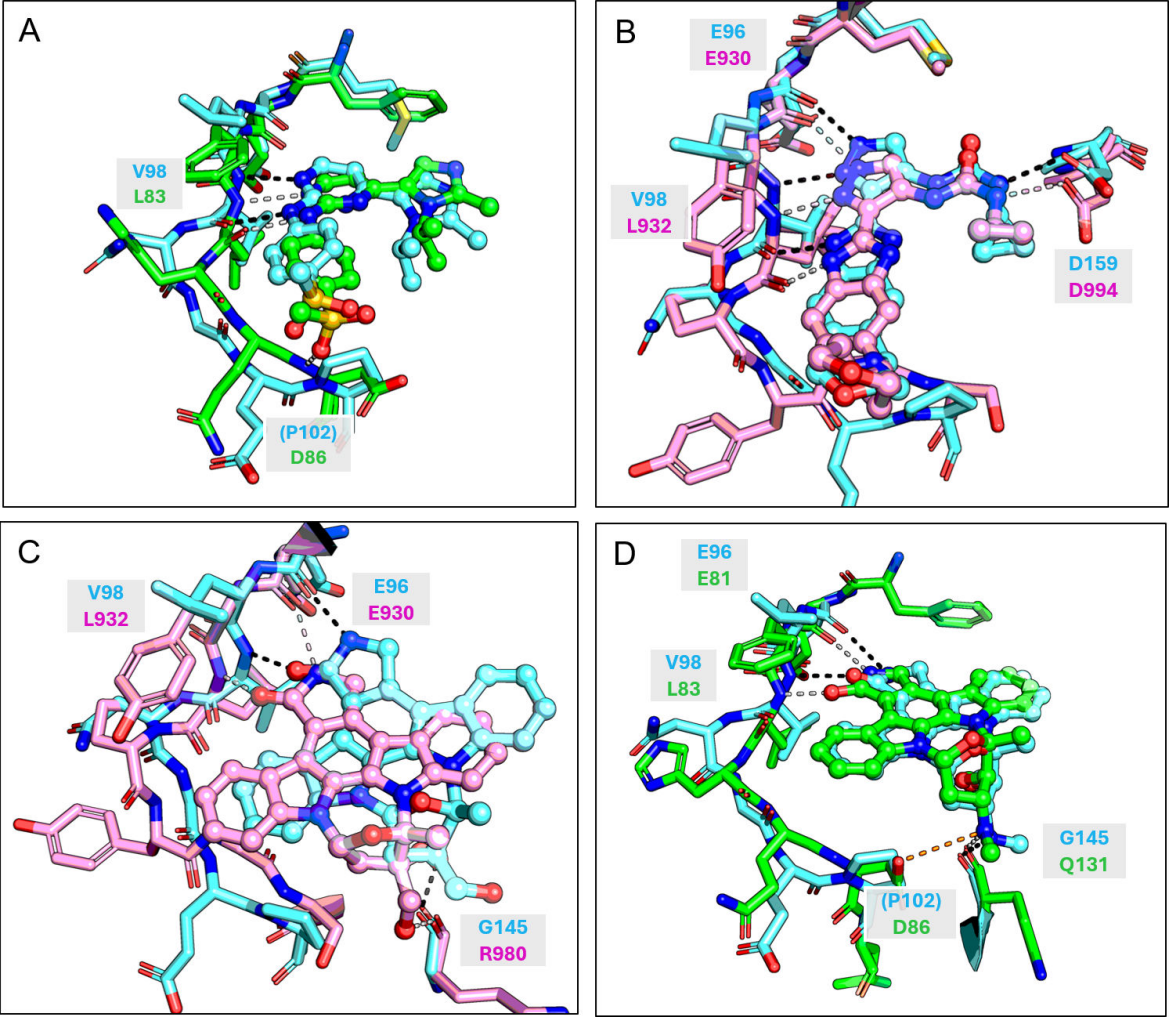
Comparison of binding modes between mycobacterial and human kinases, using available crystal structures. PknA is shown in cyan, CDK2 in green, and JAK2 in pink. Black dashed lines indicate hydrogen bonds in the PknA structures, and white dashed lines represent the hydrogen bonds in the human kinases. Non-interacting residues are shown in brackets. (**A**) Comparison of the binding of AZD-5438 to PknA and CDK2 (PDB ID: 6GUH). (**B**) Comparison of the binding of AT9283 to PknA and JAK2 (PDB ID: 9TM5). (**C**) Comparison of the binding of lestaurtinib to PknA and JAK2 (PDB ID: 8BPW). (**D**) Comparison of the binding of staurosporine to PknA and CDK2 (PDB ID: 4ERW). The orange dashed line indicates a salt bridge in CDK2.

Structural comparison of the binding of AZD-5438 between PknA and CDK2 reveals a similar binding mode between the mycobacterial and human kinase. The compound makes similar hinge interactions in both structures (Fig. 6A), despite some differences in the main-chain conformation of the hinge region. Owing to these differences, AZD-5438 forms an additional hydrogen bond in the CDK2 structure with the main-chain amide of Asp86, which is a proline in PknA (Pro102). This partly explains the low nanomolar potency of the compound toward CDK2. Comparing the binding of AT9283 to PknA or JAK2 shows a nearly identical binding pose with similar hydrogen bonding (Fig. 6B), explaining its high potency against both kinases. In the case of lestaurtinib, the compound makes the same hinge interactions with PknA and JAK2. The hydrogen bond in the catalytic loop is also found in both structures, but it is mediated by a hydroxyl group in the PknA structure and by a methoxy group in the JAK2 structure. However, in order to optimize hinge interactions, the compound shifts in the ATP-binding site leading to more efficient binding to JAK2 (Fig. 6C). Despite the same core indolocarbazole structure, staurosporine does not display a similar shift to lestaurtinib in the binding site between PknA and CDK2, and the hinge interactions are conserved between both structures (Fig. 6D). While hydrogen bonding of the positively charged N-methylammonium substituent is conserved between PknA and CDK2, the moiety makes an additional salt bridge to Asp86 at the hinge in CDK2, while the substituent is situated in a more unfavorable environment in PknA due to the presence of a proline in this position.

## DISCUSSION

Treatment of *M. tuberculosis* requires sustained administration of a combination of antibiotics over several months to eliminate the infection. To exacerbate the existing challenges, the emergence of antibiotic resistance further complicates therapy. Extensively drug-resistant infections can be fatal when effective treatment options are unavailable. To avoid resistance development, new targets in antimycobacterial drug discovery are needed. To this end, we focused on three essential mycobacterial serine/threonine kinases, PknA, PknB, and PknG, which play critical roles in bacterial survival and disease progression. These kinases share the canonical kinase fold and sufficient structural similarity, enabling multitargeting by small-molecule inhibitors to the ATP-binding site. Simultaneous inhibition of multiple essential kinases offers a promising strategy to enhance bactericidal efficacy and reduce the likelihood of resistance development that often arises with single-target therapies (37). By screening an in-house kinase inhibitor library, we identified four compounds capable of inhibiting all three kinases at micromolar concentrations, with AT9283 demonstrating submicromolar potency across all targets. Other hits were AZD-5438, lestaurtinib, and staurosporine. All hit compounds are known human kinase inhibitors.

Thermodynamic analysis demonstrated variant binding profiles for the compounds. The binding of AT9283 to PknA and PknB was exclusively driven by enthalpy, whereas PknG displayed an additional positive entropy contribution. Enthalpy-driven binding releases energy by bond formation (hydrogen bonds, electrostatic interactions, and van der Waals contacts) between the protein and ligand. Entropy-driven ligand binding occurs when ordered water is released or when hydrophobic surfaces are brought into contact. Mechanistically, the binding of all the hits is similar in PknA and PknB, which is also reflected by the higher conservation of their ATP-pockets (Fig. 1). In contrast, PknG features a more closed ATP-binding site due to an additional N-terminal segment and a less charged binding surface compared to PknA and PknB (Fig. S5). These structural differences alter the thermodynamic profile of inhibitor binding and likely complicate multitargeting strategies that include PknG alongside PknA and PknB.

An essential step in drug development is confirming that compounds can engage their intracellular targets. To assess this, we tested the inhibitors in avirulent *M. tuberculosis* cultures to determine their ability to inhibit bacterial growth. High concentrations of compounds were required for the inhibition of bacterial growth, indicating poor intracellular availability. Several factors can contribute to this outcome. First, the thick, mycolic acid-rich cell wall of *M. tuberculosis* is a well-known barrier that significantly limits passive diffusion of many compounds (20, 38). Second, the physicochemical properties such as molecular weight and lipophilicity play a critical role in permeability. Optimizing the LogP value to achieve optimal lipophilicity may enhance membrane penetration without compromising solubility. Combination strategies could also improve efficacy. As an example, co-administering ethambutol may facilitate compound entry as it disrupts cell wall integrity (20, 39). Additionally, prodrug approaches or conjugation to molecules actively imported for bacterial metabolism, such as trehalose, could circumvent permeability limitations (40). Collectively, these challenges highlight the need for rational optimization of compound properties and delivery strategies to achieve effective intracellular target engagement.

**Fig 7:**
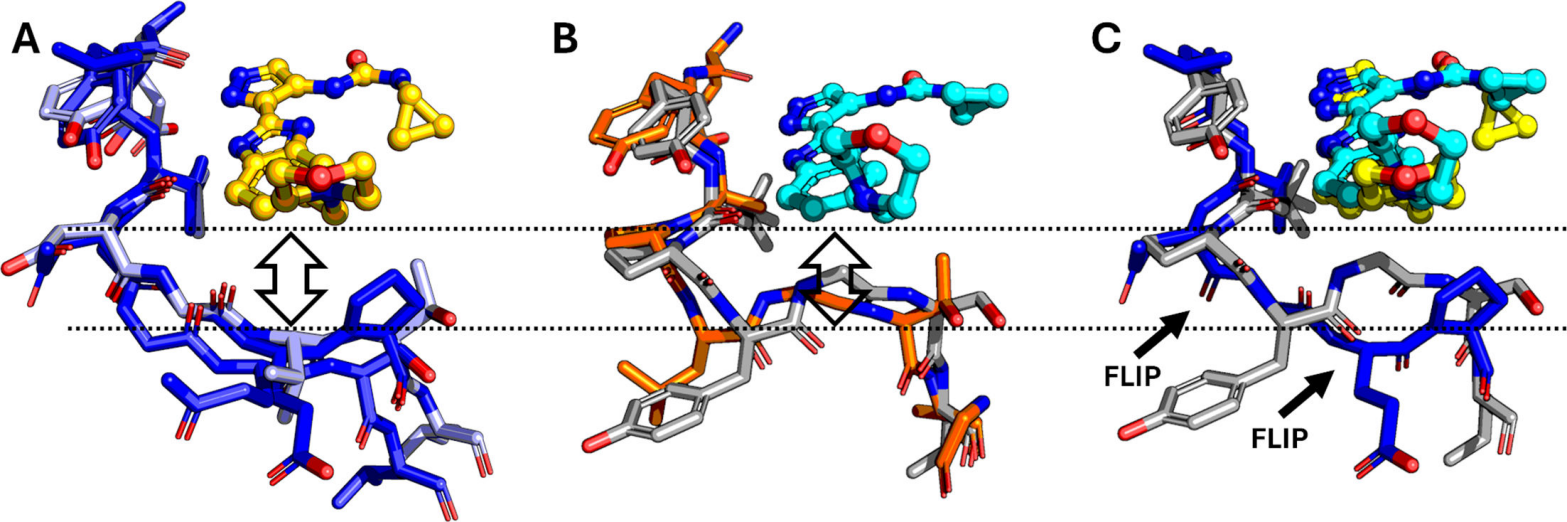
Structural basis for improved inhibitor selectivity. The hinge region of Pkn kinases differs from human kinases, and these differences can be utilized in drug design. (**A**) Superposition of PknA–AT9283 complex, PknB (PDB ID: 1O6Y), and PknG (PDB ID: 4Y0X) crystal structures (different shades of blue) show similar backbone orientation and a large distance from AT9283 (yellow). (**B**) Superposition of JAK2–AT9283 complex (gray) and Aurora A kinase (orange, PDB ID: 1MQ4) shows tight packing of the hinge against the inhibitor (cyan). (**C**) Comparison of PknA–AT9283 (gray) to JAK2–AT9283 (blue) complexes. Arrows indicate positions of main-chain flips leading to a different hinge orientation.

The human kinome consists of 518 protein kinases. Therefore, optimizing selectivity toward mycobacterial kinases is essential in preventing off-target effects during therapy. The hinge region (Fig. 1) connects the N- and C-lobes of the kinase domain, providing a binding site for ATP and an anchoring point for the majority of kinase inhibitors, influencing the binding affinities of many kinase-targeting inhibitors. Notably, the hinge regions of PknA, B, and G differ markedly from those of major human off-target kinases. Figure 7 shows the differences between the hinge regions between the Pkn kinases and two major off-target human kinases, JAK2 and Aurora-A kinase. In JAK2 and Aurora-A, AT9283 sits much closer to the hinge than it does in the Pkn kinases (Fig. 7A and B). This displacement arises from a main-chain flip in the hinge of the Pkn kinases relative to the human kinases. That conformational difference can be exploited to improve Pkn selectivity, for example, by extending inhibitors toward the flipped region so that they sterically clash with the human kinase hinge conformation while remaining compatible with the Pkn kinase hinge.

In summary, we identified multitargeting inhibitors against three mycobacterial kinases and analyzed their binding modes to facilitate the future development of anti-TB agents. Structural differences between human kinases and mycobacterial Pkn kinases, which can be used for increasing selectivity, were also identified. Poor cellular activity of the identified hits suggests that intracellular compound availability is a major issue affecting the potency of these compounds. Nonetheless, these multitargeting compounds and their detailed structural characterization represent a viable starting point for the development of potent Pkn inhibitors.

## MATERIALS AND METHODS

### Protein production and purification

PknA (1–283) and PknG (74–405) sequences were cloned in pNic28-Bsa4 vectors, and PknB (1–279) was cloned in the pET-28a(+) vector. The proteins were expressed in *Escherichia coli* BL21* (DE3) cells. The cells carrying the PknA plasmid were cultivated in TB-AIM (Formedium) supplemented with 8 g/L glycerol and 30 µg/mL kanamycin. The cells were grown at 37°C until OD_600_ reached approximately 1.0, and the temperature was lowered to 16°C for protein expression for 48 h. The cells carrying the PknB and PknG plasmids were grown in TB (Formedium) supplemented with 30 µg/mL kanamycin. The cells were grown at 37°C until OD_600_ reached 0.6–1.0. Protein expression was induced with 1 mM IPTG at 15°C for 18 h (PknB) and with 0.5 mM IPTG at 16°C for 18 h (PknG). The cells were collected by centrifugation (4,000 × *g*, 10 min) and resuspended in lysis buffer (50 mM Tris-HCl pH 8.0, 500 mM NaCl, 10% glycerol, 0.5 mM TCEP, 20 mM imidazole). The cell suspension was supplemented with 9,000 U/mL lysozyme, 20 µg/mL DNase I, 5 mM MgCl_2_, 0.1 mM PMSF, and 35 µM Polymyxin B. Cells were lysed either by five cycles of homogenization at 80 psi (Avestin Emulsiflex-C3) or by five freeze-thaw cycles in liquid nitrogen. The lysates were clarified at 30,000 × *g* at 4°C for 45 min, after which the supernatants were incubated with Ni-NTA resin (Invitrogen) at 4°C for 1 h. The resins were loaded and washed with 50 mL lysis buffer in a gravity-flow column. The proteins were eluted with lysis buffer containing 250 mM imidazole. The proteins were further purified with gel filtration using Superdex 75 16/600 (Cytiva) column equilibrated with 20 mM Tris-HCl pH 8.0, 300 mM NaCl, 10% glycerol, and 0.5 mM TCEP. The purified proteins were concentrated using 10 kDa MWCO concentrators (Millipore), aliquoted, flash-frozen with liquid nitrogen, and stored at −70°C. JAK2 was expressed and purified as described previously (34).

### Inhibitor screening with the kinase activity assay

An in-house kinase inhibitor library consisting of 236 kinase inhibitors and kinase inhibitor analogs was used for compound screening. The screening was carried out as previously described (41) using black 384-well microplates (ProxiPlate-384 F Plus, Revvity). The kinase reaction was performed in triplicate in a total volume of 5 µL. Final concentrations of reactants were 2 µM PknA, 100 µM ATP, and 100 µM compound (in assay buffer, 100 mM Tris-HCl pH 7.5, 5 mM MgCl_2_, 0.01% Tween 20, 0.01% BSA), which were allowed to react at room temperature with shaking at 300 rpm for 2 h. The amount of ADP produced by the kinase reaction was quantified by adding 5 µL of the detection solution (2 mM glucose, 200 µM NADP^+^, 100 µM resazurin, 1 µM ADP-hexokinase, 0.02 U/mL G6PDH, and 0.02 U/mL diaphorase in 100 mM Tris-HCl, pH 7.5) to the kinase reaction. After 1 h of incubation (with shaking at 300 rpm), the fluorescence was measured with excitation at 540 nm and emission at 570 nm using the Tecan Spark plate reader.

### Potency measurements with the kinase activity assay

The IC_50_ values of the compounds were measured in triplicate as described above. The compounds were diluted half-logarithmically to produce nine data points, with final concentrations ranging from 100 µM to 10 nM. The concentrations of PknA and PknB were 2 µM. The data were fitted with the non-linear regression analysis, dose-response fitting with variable slope (four-parameters), using GraphPad Prism version 10.3.1.

### Fluorescence polarization

The potency measurements were carried out in a total volume of 5 µL in 20 mM Tris-HCl (pH 8.0), 150 mM NaCl, 20% glycerol, 0.01% Brij-35, and 2 mM DTT. The half-logarithmic compound dilutions (final concentrations ranging from 100 µM to 10 nM) were prepared in triplicate and mixed with 2 µM of PknA, 1 µM of PknB or 550 nM PknG, and 1.5 nM of the fluorescently labeled tracer (JAK2 JH2 tracer, HY-102055, MedChemExpress) in black 384-well microplates (ProxiPlate-384 F Plus, Revvity). The plates were incubated at room temperature for 5 min with shaking (300 rpm), and the fluorescence polarization values were measured using the Tecan Spark plate reader with excitation at 490 nm and emission at 522 nm. For obtaining the dose-response curves, the data were fitted using non-linear regression analysis with a variable-slope (four-parameter) dose-response model in GraphPad Prism version 10.3.1.

### Isothermal titration calorimetry

The binding of the compounds to PknA, PknB, and PknG was analyzed by ITC. Titrations were performed at 25°C with 10–200 µM of compound titrated into 5–20 µM protein in ITC buffer (20 mM HEPES pH 8.0, 200 mM NaCl, 0.5 mM TCEP). The experiments were performed using VP-ITC (Malvern Panalytical Ltd., UK) and analyzed using Origin 7 (OriginLab).

### Cell-based assays

Avirulent *Mycobacterium tuberculosis* (*M. tuberculosis* H37Ra) were cultured from freezer stocks in medium (Middlebrook 7H9 supplemented with 2% [vol/vol] glycerol and 0.5% [vol/vol] Tween 80) (Middlebrook 7H9 BD #271310, Tween 80 Merck #P1754). Cultures were incubated at 37°C with slow shaking for 12 days until reaching an optical density at 650 nm (OD₆₅₀) of 0.2–0.4. The cultures were then diluted 1:100 in fresh medium and incubated under the same conditions for an additional 4–5 days until OD₆₅₀ reached approximately 0.2. For MIC assays, the cultures were further diluted 1:1,000 for liquid MIC assay in the medium. Bacteria were transferred to sterile 96-well plates. A two-fold serial dilution was performed to all tested drugs (0.01–100 μM). DMSO served as the negative control, and positive control drugs, isoniazid and rifampicin, were included in parallel using the same dilution scheme. The drug dilutions were added onto the cells. The plates were sealed with lids, placed in ziplock bags, and incubated at 37°C. After one week, bacterial growth was assessed visually. Plates were then returned to the incubator for an additional week to confirm results. MIC was determined as the lowest concentration of inhibitor that led to a visible increase in turbidity after 14 days of culture.

### Protein crystallization

PknA (10.5 mg/mL) was crystallized using the sitting-drop vapor diffusion method. The crystallization drops were equilibrated against 1.1–1.2 M ammonium sulfate and 0.1 M Bis-Tris pH 6.5 at 22°C. Microseeding with apo-PknA crystals was utilized to obtain well-diffracting crystals. PknA in complex with AZD-5438 and AT9283 was obtained via co-crystallization with 0.5 mM compound and microseeding. Lestaurtinib and staurosporine were soaked (0.5 mM lestaurtinib or 1 mM staurosporine) into apo-PknA crystals (1.2 M ammonium sulfate, 0.2 M NaCl and 0.1 M Bis-Tris, pH 6.5) for two months. The crystals were cryoprotected in well solution supplemented with 20% glycerol and flash-frozen in liquid N_2_ for data collection. JAK2 was co-crystallized with AT9283 as described previously (34).

### Data collection, structure determination, and refinement

The diffraction data were collected on the I03 beamline at Diamond Light Source (Didcot, UK). Data were processed and scaled using the XDS package (42). Molecular replacement was performed with Phaser (43) using apo-PknA (PDB ID: 4OW8) (44) or the JAK2-cerdulatinib complex (PDB ID: 8BX6) (34) as the search model. The structures were refined using phenix.refine (45). The data were visualized and manually built using Coot (46). Data processing and refinement statistics are shown in Table S2.

### Molecular docking

Molecular docking and compound preparation were performed with the Maestro package (Maestro, Schrödinger, LLC, New York, NY, 2025). Compounds were prepared with Ligprep to add hydrogen atoms, generate three-dimensional conformations and tautomers, adjust the ionization states, and minimize the energy of output structures using the OPLS4 force field (47). PknB (PDB ID: 5U94) and PknG (PDB ID: 4Y0X) structures were prepared for docking by adding hydrogens and optimizing hydrogen bonding and minimizing the structure. Water molecules were also removed prior to docking. Ligands were docked to a grid around the bound ligand in the crystal structures using Glide (48, 49).

### Structure visualization

Visualization of protein structures, structural figures, and electrostatics calculations (50) was done using PyMol 3.1.6.1.

## Data Availability

The structures for PknA in complex with the inhibitors AZD-5438, AT9283, lestaurtinib, and staurosporine have been deposited in the Protein Data Bank (https://www.rcsb.org/) under the accession codes of 9TMX, 9TN5, 9TMQ, and 9TMR, respectively. The JAK2–AT9283 complex has been deposited with an accession code of 9TM5.

## SUPPLEMENTAL MATERIAL

Supplemental figures and tables spectrum.00049-26-s0001.docx Figures S1 to S5; Tables S1 and S2.

ASM does not own the copyrights to Supplemental Material that may be linked to, or accessed through, an article. The authors have granted ASM a non-exclusive, world-wide license to publish the Supplemental Material files. Please contact the corresponding author directly for reuse.
