# Supplementary material for: Structural insights into multitargeting Mycobacterium tuberculosis Pkn kinases
Source: Microbiol Spectr. 2026 Jul 14;14(8):e00049-26. doi: 10.1128/spectrum.00049-26 (PMC13435872; doi:10.1128/spectrum.00049-26)
Supplement: Supplementary file 1 [file spectrum.00049-26-s0001.docx]

Supplemental material

**Structural Insights into Multitargeting Mycobacterium tuberculosis Pkn Kinases**

Angelika Pölläniemi^a^, Ya Miao^a^, Lotta Laitila^a^, Hannaleena Piippo^a^, Milka Hammarén^a,b^, Mataleena Parikka^a^, Teemu Haikarainen^a,c,#^

^a^Faculty of Medicine and Health Technology, Tampere University, 33520 Tampere, Finland

^b^European Molecular Biology Laboratory, Heidelberg Germany

^c^Fimlab Laboratories, 33520 Tampere, Finland

Running title: Inhibition of M. tuberculosis Pkn Kinases

^#^Correspondence: [teemu.haikarainen@tuni.fi](mailto:teemu.haikarainen@tuni.fi)

**Fig. S1**. Compound screening. The hit limit was set to 50% of PknA inhibition which is indicated with the dotted line. The hits are denoted with squares.

 **Fig. S2**. MIC results. The minimal inhibitory concentration (µM) is marked by the text ‘MIC’. Compounds diluted two-fold starting from 100 µM except for rifampicin, which was diluted from 10 µM.

**Fig. S3.** ITC results. The numbers correspond to the compounds. 1: AZD-5438, 2: AT-9283, 3: Lestaurtinib, 4: Staurosporine. **A.** Results for PknA. **B.** Results for PknB. **C.** Results for PknG.

**Fig. S4.** Protein-ligand interactions in the binding pocket. The kinase hinge is positioned on the left side of the pocket. **A.** AZD-5438 **B.** AT9283 **C.** Lestaurtinib **D.** Staurosporine

**Fig. S5.** ATP-binding pocket electrostatics. Two different views of the ATP-binding pockets of PknA, PknB and PknG with electrostatics calculations. **A, D.** Crystal structure of PknA-AT9283 complex. **B, E.** Docking of AT9283 to PknB crystal structure (PDB code 5U94). **C, F.** Docking of AT9283 to PknG crystal structure (PDB code 4Y0X). N-terminal segment in **C** leads to a more closed ATP-binding site compared to **A** and **B**. The binding pocket in **F** is less charged than in **D** and **E**. Blue: positive potential. Red: negative potential. White: Neutral potential (near zero charge; hydrophobic or uncharged regions).

**Table S1.** IC_50_ values for the main human targets of the compounds.

|  | AZD-5438 | AT9283 | Lestaurtinib | Staurosporine |
| --- | --- | --- | --- | --- |
| JAK2 | - | 1200 nM^1^ | 1 nM^2^ | Pan-kinase inhibitor |
| CDK1 | 16 nM^3^ | 1700 nM^1^ | - |  |
| CDK2 | 45 nM^3^ | 510 nM^1^ | - |  |
| CDK9 | 20 nM^3^ | - | - |  |
| AURKA | - | 3 nM (52 %)^1^ | - |  |
| AURKB | Yes^4*^ | 3 nM (58 %)^1^ | - |  |
| ABL | - | 4 nM (T315I)^1^ | - |  |
| FLT3 | - | 10 - 20 nM^1^ | 2-3 nM^4^ |  |
| TrkA | - | - | 25 nM^5^ |  |

* 94.7% mean inhibition

References

1. Howard, S. *et al.* Fragment-based discovery of the pyrazol-4-yl urea (AT9283), a multitargeted kinase inhibitor with potent aurora kinase activity. *J. Med. Chem.* **52**, 379–388 (2009).

2. Dobrzanski, P. *et al.* CEP-701 Is a JAK2 Inhibitor Which Attenuates JAK2/STAT5 Signaling Pathway and the Proliferation of Primary Cells from Patients with Myeloproliferative Disorders. *Blood* **108**, 3594 (2006).

3. Byth, K. F. *et al.* AZD5438, a potent oral inhibitor of cyclin-dependent kinases 1, 2, and 9, leads to pharmacodynamic changes and potent antitumor effects in human tumor xenografts. *Mol. Cancer Ther.* **8**, 1856–1866 (2009).

4. Knapper, S. *et al.* A phase 2 trial of the FLT3 inhibitor lestaurtinib (CEP701) as first-line treatment for older patients with acute myeloid leukemia not considered fit for intensive chemotherapy. *Blood* **108**, 3262–3270 (2006).

5. Shabbir, M. & Stuart, R. Lestaurtinib, a multitargeted tyrosinse kinase inhibitor: from bench to bedside. *Expert Opin. Investig. Drugs* https://doi.org/10.1517/13543781003598862 (2010) doi:10.1517/13543781003598862.

**Table S2.** Data collection and refinement statistics.

|  | **PknA-AZD-5438 (PDB ID 9TMX)** | **PknA- AT9283 (PDB ID 9TN5)** | **PknA-lestaurtinib (PDB ID 9TMQ)** |
| --- | --- | --- | --- |
| **Data** |  |  |  |
| Beam line | Diamond I03 | Diamond I03 | Diamond I03 |
| Wavelength (Å) | 0.97625 | 0.97625 | 0.97625 |
| Space group | I2 | I2 | I2 |
| Cell dimensions |  |  |  |
| a, b, c (Å) | 57.96, 58.81, 73.48 | 107.88, 69.30, 50.24 | 57.98, 58.28, 72.66 |
| α, β, γ (°) | 90.00, 96.09, 90.00 | 90.00, 99.19, 90.00 | 90.00, 97.32, 90.00 |
| Resolution (Å) | 47.8-1.70 (1.73-1.70) | 58.1-1.45 (1.48-1.45) | 48-2.13 (2.19-2.13) |
| CC_1/2_ (%) | 99.9 (52.6) | 99.9 (39.2) | 99.1 (25.0) |
| *I/σI* | 16.5 (1.1) | 13.0 (1.9) | 4.8 (1.3) |
| *R*_merge_ | 0.072 (1.857) | 0.097 (1.007) | 0.253 (1.383) |
| Completeness (%) | 99.5 (99.9) | 99.9 (99.7) | 99.6 (99.9) |
| Redundancy | 7.0 (7.2) | 6.9 (7.0) | 6.9 (7.0) |
| **Refinement** |  |  |  |
| Reflections | 26952 | 28988 | 13502 |
| R_work_/R_free_ | 0.212/ 0.227 | 0.214/0.230 | 0.292/0.349 |
| RMSD of bond lengths (Å) | 0.006 | 0.004 | 0.007 |
| RMSD of bond angles (°) | 0.647 | 0.719 | 1.202 |
| *B*-factors (Å^2^) |  |  |  |
| Protein | 37.2 | 36.7 | 43.1 |
| Ligand | 26.4 | 14.2 | 28.8 |

Values for the highest resolution shell are shown in parentheses.

**Table S2.** Data collection and refinement statistics. Continued.

|  | **PknA-staurosporine (PDB ID 9TMR)** | **JAK2-AT9283 (PDB ID 9TM5)** |
| --- | --- | --- |
| **Data** |  |  |
| Beam line | Diamond I03 | Diamond I03 |
| Wavelength (Å) | 0.97625 | 0.97625 |
| Space group | I2 | C2 |
| Cell dimensions |  |  |
| a, b, c (Å) | 57.86, 58.29, 71.32 | 107.88, 69.30, 50.24 |
| α, β, γ (°) | 90.00, 98.92, 90.00 | 90.00, 99.19, 90.00 |
| Resolution (Å) | 48.2-1.90 (1.94-1.90) | 58-1.45 (1.48-1.45) |
| CC_1/2_ (%) | 99.9 (34.3) | 99.9 (39.2) |
| *I/σI* | 9.6 (0.7) | 13.0 (1.9) |
| *R*_merge_ | 0.119 (2.353) | 0.097 (1.007) |
| Completeness (%) | 97.5 (90.5) | 99.9 (99.7) |
| Redundancy | 7.1 (6.7) | 6.9 (7.0) |
| **Refinement** |  |  |
| Reflections | 18048 | 64639 |
| R_work_/R_free_ | 0.248/ 0.277 | 0.179/0.198 |
| RMSD of bond lengths (Å) | 0.018 | 0.007 |
| RMSD of bond angles (°) | 1.483 | 1.101 |
| *B*-factors (Å^2^) |  |  |
| Protein | 42.6 | 24.9 |
| Ligand | 28.1 | 28.6 |

Values for the highest resolution shell are shown in parentheses.
